# dTACC restricts bouton addition and regulates microtubule organization at the *Drosophila* neuromuscular junction

**DOI:** 10.1002/cm.21578

**Published:** 2019-11-21

**Authors:** Vivian T. Chou, Seth Johnson, Jennifer Long, Maxime Vounatsos, David Van Vactor

**Affiliations:** ^1^ Department of Cell Biology and Program in Neuroscience Blavatnik Institute, Harvard Medical School Boston Massachusetts

**Keywords:** *Drosophila melanogaster*, microtubules, synapse, transforming acidic coiled coil protein

## Abstract

Regulation of the synaptic cytoskeleton is essential to proper neuronal development and wiring. Perturbations in neuronal microtubules (MTs) are associated with numerous pathologies, yet it remains unclear how changes in MTs may be coupled to synapse morphogenesis. Studies have identified many MT regulators that promote synapse growth. However, less is known about the factors that restrict growth, despite the potential links of synaptic overgrowth to severe neurological conditions. Here, we report that dTACC, which is implicated in MT assembly and stability, prevents synapse overgrowth at the *Drosophila* neuromuscular junction by restricting addition of new boutons throughout larval development. dTACC localizes to the axonal MT lattice and is required to maintain tubulin levels and the integrity of higher‐order MT structures in motor axon terminals. While previous reports have demonstrated the roles of MT‐stabilizing proteins in promoting synapse growth, our findings suggest that in certain contexts, MT stabilization may correlate with restricted growth.

## 1 INTRODUCTION

Synapses are the essential functional units of the nervous system. Formation of intricate synaptic geometry, which involves complex arborization of cell processes and cell–cell connections, is critical to the function and plasticity of neural circuits. Following axon pathfinding, signaling pathways coordinate synaptic morphogenesis and the formation of stable junctions between pre‐ and postsynaptic compartments (Collins & DiAntonio, 2007; Goda & Davis, 2003; Van Vactor & Sigrist, 2017). A major target and effector of these signaling networks is the presynaptic microtubule (MT) cytoskeleton (Broadie & Richmond, 2002; Menon, Carrillo, & Zinn, 2013; Ruiz‐Cañada & Budnik, 2006). MTs have been linked to numerous neurodevelopmental and neurodegenerative disorders (Bodaleo & Gonzalez‐Billault, 2016; Goellner & Aberle, 2012; Lasser, Tiber, & Lowery, 2018; Matamoros & Baas, 2016). However, despite the clear importance of synaptic MTs, our understanding of their regulation and function still lags behind our comprehension of the upstream signaling pathways that orchestrate synapse development.

Despite limited mechanistic understanding, synaptic morphogenesis has been well characterized at the phenomenological level through studies at the *Drosophila* neuromuscular junction (NMJ; Jan & Jan, 1976). In this system, a motor axon contacts its target muscle during late embryogenesis and transitions from a motile, sheet‐like growth cone into a branched structure decorated with synaptic varicosities (“boutons”; Yoshihara, Rheuben, & Kidokoro, 1997). Throughout larval development, the NMJ rapidly expands through the addition of new immature boutons (Schuster, Davis, Fetter, & Goodman, 1996; Zito, Parnas, Fetter, Isacoff, & Goodman, 1999), which then recruit presynaptic active zone components and postsynaptic receptors as they mature (Menon et al., 2013; Vasin et al., 2014). In response to both developmental cues and neural activity (Budnik, Zhong, & Wu, 1990; Chklovskii, Mel, & Svoboda, 2004; Van Vactor & Sigrist, 2017), the NMJ undergoes continuous remodeling via both bouton addition and removal (Eaton, Fetter, & Davis, 2002; Fuentes‐Medel et al., 2009). These processes are modulated by numerous signaling pathways, such as BMP (Bayat, Jaiswal, & Bellen, 2011; Keshishian & Kim, 2004), FGF (Sen et al., 2011), LAR (Han, Jeon, Um, & Ko, 2016; Um & Ko, 2013), and Wnt/Wg (Packard et al., 2002; Park & Shen, 2012; Speese & Budnik, 2007).

While the downstream MT‐related targets of developmental signaling pathways remain largely unknown, several components of the Wnt/Wg pathway directly regulate the MT cytoskeleton by binding MTs themselves and/or with MT‐associated proteins (MAPs; Salinas, 2007). One such target of Wnt/Wg signaling is the MAP Futsch (homolog of MAP1B), which is phosphorylated by glycogen synthase kinase 3 (GSK3)/Shaggy (Sgg) in both mammals and flies (Cohen & Frame, 2001; Franco et al., 2004; Gögel, Wakefield, Tear, Klämbt, & Gordon‐Weeks, 2006). At the *Drosophila* NMJ, Futsch promotes MT stability and synaptic expansion (Lepicard, Franco, de Bock, & Parmentier, 2014; Roos, Hummel, Ng, Klämbt, & Davis, 2000), while inhibition of Futsch by Sgg restricts synapse size (Franco et al., 2004). These findings suggest a model where increased stabilization of MTs is associated with increased NMJ expansion. Additional factors that are associated with MT stability, such as the formins Diaphanous (Pawson, Eaton, & Davis, 2008) and DAAM (Bartolini & Gundersen, 2010; Migh et al., 2018), have also been found to promote NMJ expansion.

As a counterbalance to MT‐stabilizers, MT destabilizers/severing proteins, such as Spastin (Sherwood, Sun, Xue, Zhang, & Zinn, 2004) and Katanin (Mao et al., 2014) restrict NMJ size. Consistently, repression of Futsch mRNA levels by Dfxr (homolog of FMR1) prevents NMJ overgrowth (Zhang et al., 2001), further supporting the notion that MT stability correlates with NMJ expansion. Interestingly, mutation of human *spastin* is the most frequent cause of hereditary spastic paraplegias (HSP; Solowska & Baas, 2015), while mammalian *katanin* has been associated with behavioral deficits and intellectual disability (Banks et al., 2018; Bartholdi et al., 2014). Similarly, *dfxr* is associated with Fragile X syndrome, one of the most common forms of inherited intellectual disability (Penagarikano, Mulle, & Warren, 2007). The synaptic phenotypes and disease relevance of genes such as *spastin*, *katanin*, and *dfxr* has led to the understanding that excessive synaptic growth is highly detrimental. Collectively, these findings suggest that a complex set of factors is responsible for maintaining a precise balance of both synapse expansion and restriction to ensure neurological function and health.

Here, we report a new negative regulator of synapse growth, the *Drosophila* homolog of the highly conserved TACC (transforming acidic coiled coil) family (Ding et al., 2017; Hood and Royle, 2011; Peset and Vernos, 2008; Thakur et al., 2013). Early studies of *Drosophila* and mammalian TACC‐family proteins showed that these proteins are often concentrated near MT minus ends and have roles in regulating MTs and spindle function during mitosis (Gergely et al., 2000; Gergely, Kidd, Jeffers, Wakefield, & Raff, 2000), in cooperation with the MT polymerase ch‐TOG/XMAP215/Minispindles (Msps; Akhmanova & Steinmetz, 2008, 2015; Brouhard et al., 2008; Lee, Gergely, Jeffers, Peak‐Chew, & Raff, 2001). Similar observations have since been reported across phyla (Bellanger & Gönczy, 2003; Le Bot, Tsai, Andrews, & Ahringer, 2003; Peset et al., 2005; Samereier, Baumann, Meyer, & Gräf, 2011; Sato, Vardy, Angel Garcia, Koonrugsa, & Toda, 2004; Srayko, Quintin, Schwager, & Hyman, 2003). TACC can also localize at the MT plus‐end, where it is thought to regulate MT assembly dynamics (Long et al., 2013; Lucaj et al., 2015; Nwagbara et al., 2014; Rutherford et al., 2016; Samereier et al., 2011; Srayko et al., 2003). However, TACC localization to the MT lattice has been observed in multiple settings (Gergely, Kidd, et al., 2000; Peset et al., 2005; Sato et al., 2004; Thadani, Ling, & Oliferenko, 2009). Altogether, these studies strongly suggest that TACC proteins serve as conserved mediators of both the assembly and stability of MTs (Ding et al., 2017; Hood and Royle, 2011; Peset and Vernos, 2008; Thakur et al., 2013).

Given (a) prior studies suggesting that increased MT stability correlates with growth and (b) the established roles of TACC proteins in MT assembly and stability, dTACC would naturally be expected to promote synapse growth. Surprisingly, we discovered instead that presynaptic dTACC negatively regulates the growth of the larval NMJ by limiting addition of synaptic boutons during development. We also found that within the motor axon terminal, dTACC associates abundantly along the lattice of MTs and regulates both the integrity and higher‐order organization of MTs. Our results suggest that in certain contexts, assembly and/or organization of MTs by proteins such as dTACC may restrict NMJ expansion.

## 2 RESULTS AND DISCUSSION

### 2.1 Presynaptic dTACC is required to restrict NMJ size

**Figure 1:**
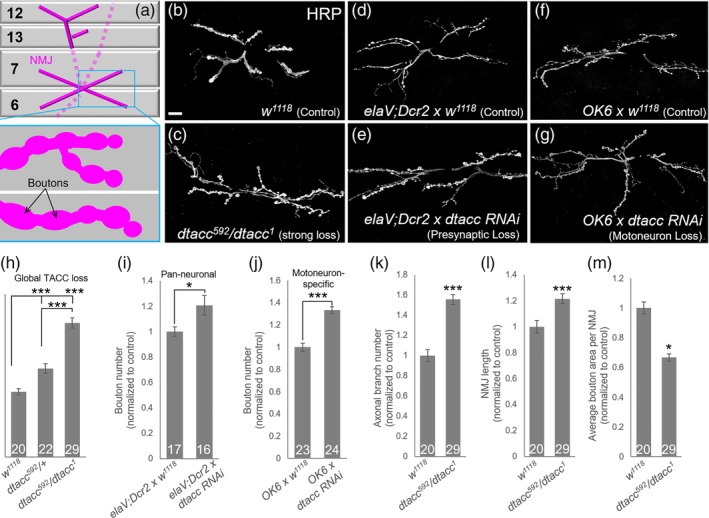
dTACC is a negative regulator of NMJ size. (a) Schematic of larval ventral musculature and nerve innervation pattern. For clarity, only select structures are depicted. Inset shows the morphology of the NMJ, including varicosities or “boutons.” (b–j) Loss of dTACC results in NMJ overgrowth. (b–g) Images show third instar NMJs stained with the neuronal membrane marker α‐HRP. In contrast to *w* ^*1118*^ controls (b), *dtacc* ^*592*^ */dtacc* ^*1*^ flies (c) showed increased NMJ size, as did *elaV* ^*C155*^; *Dcr2 x dtacc‐RNAi* (e) and *OK6 x dtacc‐RNAi* (g) animals compared to their respective *elaV* ^*C155*^; *Dcr2 x w* ^*1118*^ (d) and *OK6 x w* ^*1118*^ (f) controls. Quantification of bouton number (h) indicates that dTACC is haploinsufficient, as *dtacc* ^*592*^ */+* heterozygotes show a 1.35‐fold increase in bouton number, while *dtacc* ^*592*^ */dtacc* ^*1*^ flies show a more severe, albeit qualitatively indistinguishable, 2.05‐fold increase. *elaV* ^*C155*^; *Dcr2 x dtacc‐RNAi* (i) and *OK6 x dtacc‐RNAi* (j) animals showed 1.21‐ and 1.33‐fold increase, respectively, in bouton number, comparable to *dtacc* ^*592*^ */+* heterozygotes. Relative to controls, *dtacc* ^*592*^ */dtacc* ^*1*^ also showed increased axonal branch number (k) and NMJ length (l). (m) *dtacc* ^*592*^ */dtacc* ^*1*^ NMJs showed a decrease in average bouton area. Raw bouton counts: *w* ^*1118*^, 96.6; *dtacc* ^*592*^ */+*, 167.6; *dtacc* ^*592*^ */dtacc* ^*1*^, 198.6; *elaV* ^*C155*^; *Dcr2 x w* ^*1118*^, 144.8; *elaV* ^*C155*^; *Dcr2 x dtacc‐RNAi*, 174.9; *OK6 x w* ^*1118*^, 127.1; *OK6 x dtacc‐rnai*, 169.4. *p < .05, ***p < .001, *t*‐test; error bars indicate ± *SEM*; number of NMJs quantified indicated on graph; scale bar, 20 μm

The NMJ has a highly stereotyped morphology consisting of a branched motor axon terminal that is decorated by numerous presynaptic boutons (Figure 1a). In this system, boutons are often quantified as a measure of overall NMJ size. To determine the effect of dTACC on synapse morphogenesis, we initially counted mature type I boutons at the muscle 6/7 NMJ in late‐stage third instar *dtacc* mutant larvae labeled with the neuronal membrane marker anti‐horseradish peroxidase (HRP; Jan & Jan, 1982). To generate a strong *dtacc* loss background, we raised transheterozygotes using two independently derived alleles: *dtacc* ^*592*^ (*d‐tacc* ^*stella*^), a complete null (Lee et al., 2001), and *dtacc* ^*1*^ (*d*‐*TACC* ^*1*^), which has been previously described as a very strong allele (Gergely, Kidd, et al., 2000). We combined *dtacc* ^*592*^ and *dtacc* ^*1*^ over *Df(3R)110*, a deletion at the locus (Figure S1A) and confirmed that these animals showed phenotypes comparable to *dtacc* ^*592*^/*dtacc* ^*1*^ transheterozygotes (Figure 1). However, *dtacc* ^*592*^/*Df(3R)110* and *dtacc* ^*1*^/*Df(3R)110* animals were very weak, suggesting that haploinsufficiencies uncovered by the deletion contributed to pleiotropy. Thus, we focused our remaining analysis on *dtacc* ^*592*^/*dtacc* ^*1*^ transheterozygotes to avoid additional phenotypes resulting from deletion of flanking genes.

Our analysis showed that *dtacc* ^*592*^/*dtacc* ^*1*^ animals display striking NMJ overgrowth compared to genetically matched *w* ^*1118*^ controls (Figure 1b,c,h); this phenotype was reminiscient of mutations in MT destabilizers such as *spastin* (Sherwood et al., 2004) and *katanin* (Mao et al., 2014). We also found that dTACC is haploinsufficient, as *dtacc* ^*592*^ */+* heterozygotes showed a significant and reproducible 1.35‐fold increase in bouton number compared to controls (Figure 1h), revealing that the NMJ is highly sensitive to levels of dTACC. As expected, *dtacc* ^*592*^ */dtacc* ^*1*^ animals displayed an even more dramatic but qualitatively comparable phenotype, including a 2.05‐fold increase in bouton number compared to controls (Figure 1b,c,h). There is thus a proportional relationship between dTACC levels and bouton number, suggesting that dTACC expression or activity could modulate NMJ expansion. Notably, the *dtacc* ^*592*^ */dtacc* ^*1*^ overgrowth phenotype was apparent as early as in first‐instar larvae (Figure S1B–D), indicating a continuous requirement for dTACC throughout the span of NMJ development. To determine if the change in neuronal structure reflects a presynaptic requirement for dTACC, we drove pan‐neuronal and motoneuron‐specific RNAi knockdown of a *UAS‐dtacc‐RNAi* construct using *elaV* ^*C155*^ and *OK6*‐GAL4 drivers, respectively. Both *elaV* ^*C155*^ (Figure 1d,e,i) and *OK6* (Figure 1f,g,k) driven RNAi showed significant overgrowth, indicating that dTACC is required presynaptically, and, more specifically, in motoneurons. The fold increase in bouton number in *dtacc‐RNAi* animals was comparable to that observed in *dtacc* ^*592*^ */+* heterozygotes (Figure 1h), likely reflecting the partial efficacy of the RNAi knockdown.

**Figure 2:**
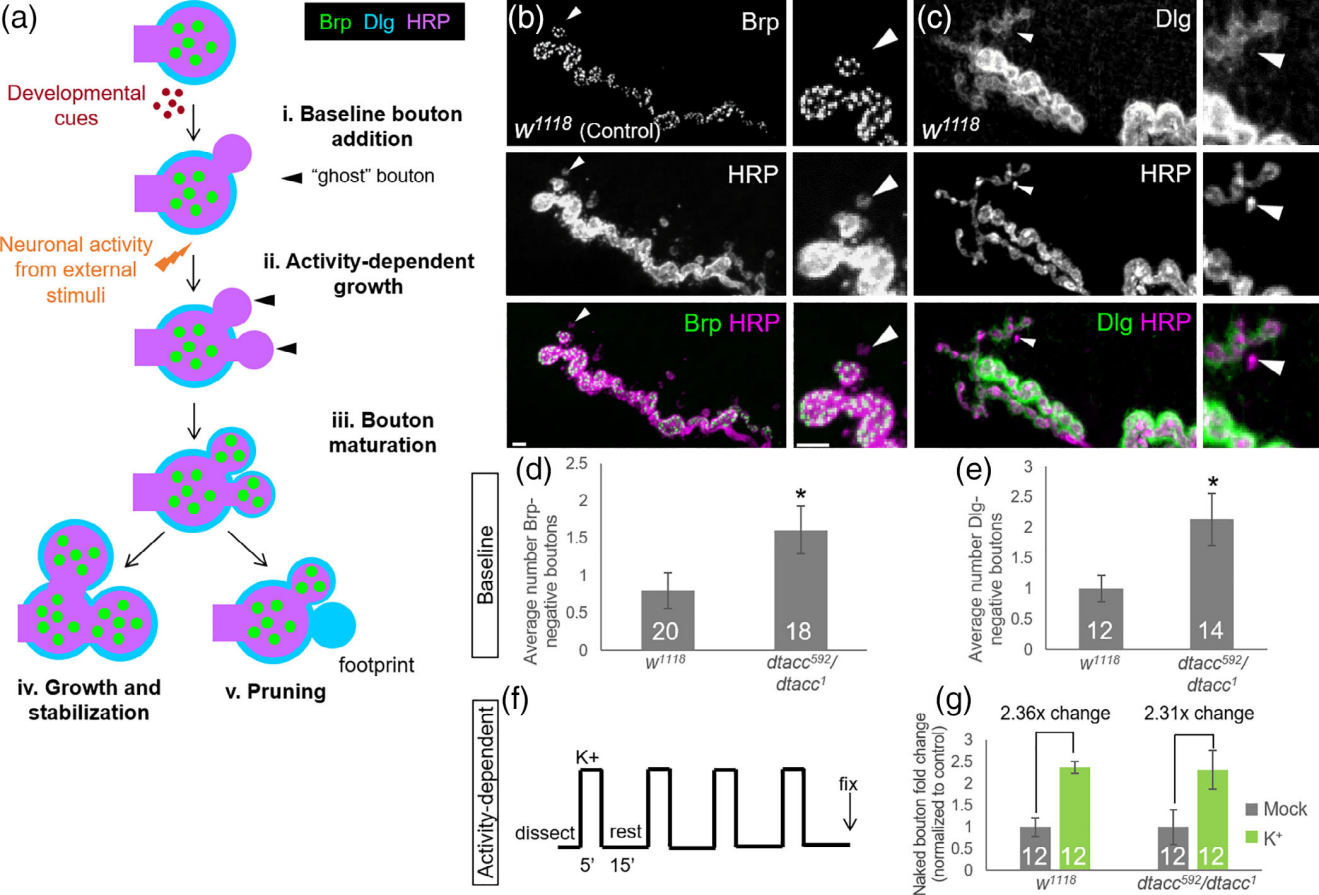
dTACC regulates bouton addition during development. (a) Schematic showing the steps of bouton formation, including addition of boutons in response to (i) baseline or (ii) activity‐dependent growth, followed by (iii) bouton maturation via recruitment of pre‐ and postsynaptic components. Boutons may continue to (iv) grow to full size and stabilize or (v) retract instead, leaving “footprints” of postsynaptic material. (b and c) NMJ stained α‐HRP and counterstained with α‐Brp (Nc82; b) or α‐Dlg (4F3; c). Nascent boutons (triangle) can be identified by the lack of postsynaptic markers such as Dlg. (d and e) *dtacc* ^*592*^ */dtacc* ^*1*^ flies showed a twofold increase in Brp‐negative boutons (d) and in Dlg‐negative boutons (e) compared to *w* ^*1118*^ controls. (f) Spaced‐stimulation paradigm used to induce rapid activity‐dependent bouton budding. (g) controls and *dtacc* ^*592*^ */dtacc* ^*1*^ showed nearly identical fold‐changes (2.36‐ and 2.31‐fold, respectively) in the number of nascent boutons following activity compared to mock‐treated controls. *p < .05, *t*‐test; error bars indicate ± *SEM*; number of NMJs quantified indicated on graph; scale bar, 1 μm

In the *dtacc* ^*592*^/*dtacc* ^*1*^ background, we also quantified branching within the motor axon terminal, overall length of the NMJ, and bouton size; both branch number and NMJ length confirmed highly significant increases of NMJ size in *dtacc* ^*592*^/*dtacc* ^*1*^ compared to control (Figure 1k,l). We found that *dtacc* ^*592*^/*dtacc* ^*1*^ animals display a ~33% decrease in average bouton area (Figure 1m). This bouton size phenotype, along with the *dtacc* overgrowth phenotype, raised the question of which step(s) of bouton formation may require dTACC. During normal NMJ expansion (Zito et al., 1999), baseline bouton addition is controlled by developmental signaling cues (Figure 2a,i), which coordinate and balance neuronal expansion with muscle growth (reviewed by Van Vactor & Sigrist, 2017). Bouton addition can also be induced acutely by activity‐dependent cues in response to stimuli (Figure 2a,ii; Budnik et al., 1990; Chklovskii et al., 2004). Immediately following baseline or activity‐induced addition, nascent boutons lack pre‐ and postsynaptic markers and thus have a “ghost”‐like appearance (Figure 2ai,ii, black triangles; Ataman et al., 2006). However, this is a highly transient state, as components required to form the presynaptic active zone and postsynaptic cytomatrix begin to accumulate within ~30 min of new bouton formation (Figure 2a,iii; Vasin et al., 2014). Following maturation, boutons may continue to grow to full size and stabilize (Figure 2a,iv). Alternatively, boutons can be removed or pruned, occasionally leaving visible “footprints” of postsynaptic material, such as the scaffold protein Discs Large (Dlg; Figure 2a,v; Eaton et al., 2002).

We investigated the potential involvement of dTACC in each of these steps. We considered the possibility that dTACC promotes bouton pruning (Figure 2a,v) but did not find changes in Dlg footprints in *dtacc* animals, making this explanation unlikely (see Section 3). Furthermore, we found no striking defects in the overall distributions of synaptic cytomatrix antigens in *dtacc* animals, such as the core active zone component Bruchpilot (Brp; Figure S2A,B) or Dlg (Figure S2C,D), suggesting that there were no catastrophic effects on initial bouton maturation (Figure 2a,iii). However, the ~33% decrease in average bouton area (Figure 1e) did suggest a defect at the stage where maturing boutons grow to full size (Figure 2a,iv). Reduction in bouton growth could reflect some compensation for the effects of overgrowth. Alternatively, this could arise because synthesis and/or transport may not increase at the same rate as bouton number, thus making materials too sparse to form normal‐sized boutons. In either scenario, the defects in bouton growth and number raised the possibility that there might be upstream defects in bouton initiation.

### 2.2 dTACC regulates bouton initiation in response to baseline developmental cues

To investigate the potential role of dTACC in bouton addition, we looked for changes in the incidence of nascent boutons, which can be identified by the lack of maturation markers such as Brp (Figure 2b) or Dlg (Figure 2c). Compared to *w* ^*1118*^ controls (mean = 0.8 boutons/NMJ), *dtacc* ^*592*^ */dtacc* ^*1*^ animals showed a twofold increase in the number of Brp‐negative nascent boutons (mean = 1.6 boutons/NMJ, *p* = .04; Figure 2d). Consistent with the Brp presynaptic marker, Dlg staining revealed that compared to controls (mean = 1 bouton/NMJ), *dtacc* animals also showed a twofold increase in small nascent boutons lacking postsynaptic specializations (mean = 2.1 boutons/NMJ, *p* = .008; Figure 2e). Collectively, these results revealed an increased frequency of “ghost” boutons in *dtacc* animals, which suggested a greater rate of bouton initiation.

The increased bouton addition in *dtacc* animals observed through end‐point analysis could occur in response to baseline developmental cues (Figure 2a,i) and/or to neural activity from external stimuli (Figure 2a,ii). To evaluate these scenarios, we tested the requirement of dTACC in acute activity‐dependent growth using a spaced‐stimulation paradigm (Figure 2f) that induces rapid budding of “ghost” boutons (Ataman et al., 2008; Nesler et al., 2013; Piccioli & Littleton, 2014; Vasin et al., 2014). Both *w* ^*1118*^ and *dtacc* ^*592*^ */dtacc* ^*1*^ animals showed an increase in Dlg‐negative nascent boutons upon stimulation, and the fold increase was indistinguishable between controls and *dtacc* animals (2.36‐ and 2.31‐fold, respectively; Figure 2g). These results indicate that dTACC is not required for acute activity‐dependent bouton initiation. This suggests that the greater frequency of nascent boutons in *dtacc* animals likely reflects an increase in baseline bouton addition in response to developmental cues. This potential role of dTACC as a negative regulator of baseline bouton addition is consistent with the observation that significant NMJ overgrowth can be observed throughout the span of development (Figure S1B–D). Moreover, the twofold increase in putative nascent boutons in *dtacc* animals (Figure 2) is equal to the twofold increase we previously observed in mature bouton number (Figure 1). This doubling of both nascent and total bouton numbers further supports the model that increased bouton number in *dtacc* mutants is due to increased baseline bouton addition.

### 2.3 dTACC colocalizes with the lattice of synaptic MTs

To better understand how dTACC might affect NMJ growth, we asked if dTACC associates with MTs at the NMJ. While TACC has been most frequently reported to localize to MT minus‐ or plus‐ends (Ding et al., 2017; Hood and Royle, 2011; Peset and Vernos, 2008; Thakur et al., 2013), dTACC puncta have been observed along the lattice of spindle MTs (Gergely, Kidd, et al., 2000). Furthermore, *Xenopus* TACC3, the TACC isoform most highly expressed in the *Xenopus* embryonic nervous system (Rutherford et al., 2016; Tessmar, Loosli, & Wittbrodt, 2002), abundantly decorates the length of MTs in egg extracts (Peset et al., 2005), and the *Schizosaccharomyces pombe* TACC homolog Alp7/Mia1p is found along MTs both in vivo (Sato et al., 2004) and in vitro (Thadani et al., 2009).

**Figure 3:**
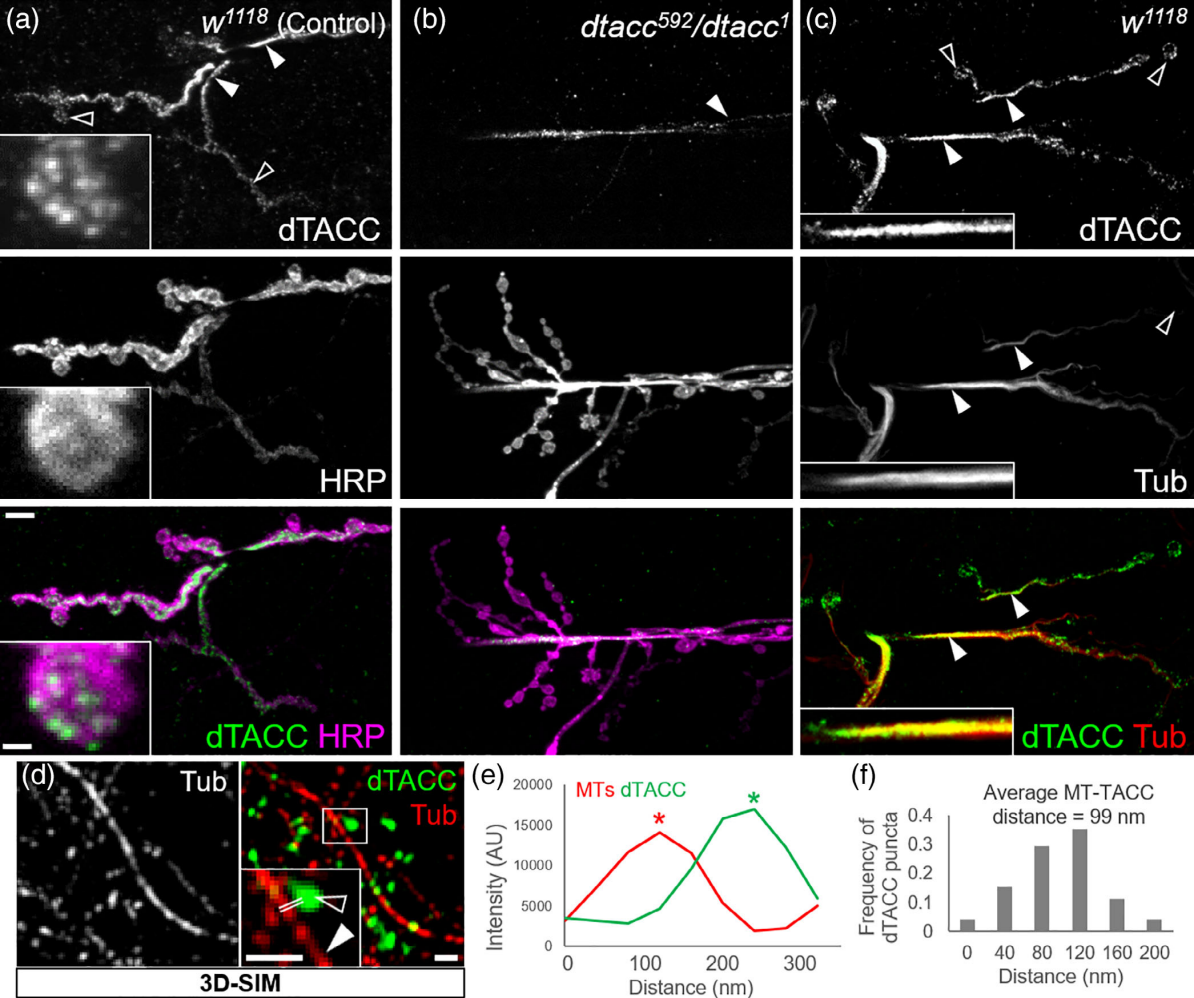
TACC colocalizes with tubulin at the synaptic terminal. (a and b) Validation of dTACC antibody in NMJs stained with α‐dTACC and α‐HRP. Compared to control *w* ^*1118*^ flies (a), *dtacc* ^*592*^ */dtacc* ^*1*^ flies showed reduced dTACC staining (b). In controls (a), the majority of TACC showed a filamentous distribution highly reminiscent of the MT lattice within motor axon terminals (solid triangles), while a smaller TACC population formed puncta (hollow triangles; see high magnification inset). In *dtacc* ^*592*^ */dtacc* ^*1*^ animals (b), punctate TACC was virtually absent, and filamentous TACC was dramatically reduced. (c) Colocalization of α‐dTACC and α‐alpha‐tubulin (Ab15246) in the axon terminal. The distribution of filamentous TACC was similar to the distribution of tubulin, while punctate TACC appeared spatially distinct from tubulin. The Manders' overlap coefficients were M1 = 56.3% ± 3.7 (percent TACC colocalizing with tubulin) and M2 = 58.6% ± 6.8 (percent tubulin colocalizing with TACC; *n* = 15 NMJs). Scale bar, 5 μm for main panels, 1 μm for insets. (d–f) Spatial relationships between punctate dTACC and MTs were examined through 3D‐SIM of samples stained with α‐dTACC and α‐alpha‐tubulin (d). Double white lines (d) represent positions from which intensity profile plots were drawn. (e) Representative intensity profile plot showing separation between the peaks of α‐dTACC and α‐tubulin staining. (f) Quantification of mean distance between peaks of α‐dTACC and α‐tubulin staining measured from intensity profile plots (*n* = 71 puncta). Mean dTACC‐MT distance was found to be 98.6 nm. Scale bar, 500 nm

We investigated the precise nature of dTACC protein localization at the NMJ to distinguish between potential modes of MT interaction in vivo. We generated and validated a novel monoclonal antibody against dTACC (see Section 3). Quantification of dTACC intensity and Western blotting confirmed loss of signal in *dtacc* mutants (Figure S3A,B). Using this antibody, we found that the majority (~90%) of dTACC signal in controls was found in polymer lattice‐like structures within the axon terminal (Figure 3A; solid triangles) which strongly resemble the MT lattice at the core of the axon, consistent with prior reports that *Caenorhabditis elegans* TAC‐1 localizes to the axons of sensory neurons (Chen et al., 2015). A smaller fraction of dTACC intensity (~10%) was found in punctate bouton‐associated structures (Figure 3a; hollow triangles). When we used our antibody on *dtacc* ^*592*^ */dtacc* ^*1*^ animals, dTACC signal was dramatically reduced with the minor punctate fraction virtually abolished and the major lattice‐like staining markedly decreased (Figure 3b). Although dTACC was not detectable on a Western blot with our antibody (Figure S3A), there was small residual immunohistochemical signal in the *dtacc* ^*592*^ */dtacc* ^*1*^ mutant (Figure S3B) that may reflect residual expression (as much as 5% in *dtacc* ^*592*^ */dtacc* ^*1*^) predicted by previous characterization of the *dtacc* ^*1*^ background (Gergely, Kidd, et al., 2000). Motivated by our finding that dTACC is required throughout early NMJ development (Figure S1B–D), we also examined dTACC distribution in first‐instar larvae (Figure S3C) and found, as expected, that dTACC is highly expressed in the ventral nerve cord (VNC) of the CNS (Figure S3D) and throughout motor/sensory axon tracts (Figure S3E).

Importantly, we tested the colocalization of dTACC with MTs by staining for alpha‐tubulin (Ab15246 for all tubulin immunohistochemistry) and found that the distribution of lattice‐like TACC and alpha‐tubulin was highly coincident (Figure 3c). The Manders' overlap coefficient M1 (percent dTACC colocalizing with tubulin) was found to be 56.3% ± 3.7, while M2 (percent tubulin colocalizing with dTACC) was 58.6% ± 6.8 (*n* = 15 NMJs; Manders, Verbeek, & Aten, 1993). We also considered the possibility that the punctate fraction of dTACC might be associated with MT plus‐ends within the motor terminal. To test this idea, we used 3‐dimensional structured illumination microscopy (3D‐SIM) to measure the average distance between dTACC and MTs based on a published methodology (Lepicard et al., 2014). Compared to confocal microscopy, 3D‐SIM improves resolution by twofold in all three dimensions and can thus resolve objects with up to eightfold smaller volume (Gustafsson, 2000; Gustafsson et al., 2008; Schermelleh, Heintzmann, & Leonhardt, 2010). At this improved resolution, we noted that dTACC puncta appeared visually distinct from synaptic MTs (Figure 3d). To confirm this observation, we generated intensity profile plots (Figure 3e) of dTACC and tubulin staining (Figure 3d, double lines show sample line scans) and found that the mean distance between the dTACC and tubulin peaks was 98.6 nm (Figure 3f). Although this method may not be sensitive enough to detect single MTs or MTs that are highly dynamic and/or labile, we were unable conclude that the distal puncta of dTACC in boutons are closely associated with MTs.

Overall, our results suggested that in the NMJ arbor, the majority of dTACC is spatially localized with the lattice of MTs, similar to prior observations in *Xenopus*, *Drosophila* and fission yeast (Gergely, Kidd, et al., 2000; Peset et al., 2005; Sato et al., 2004; Thadani et al., 2009). Interestingly, purified yeast Alp7 localizes to regions of overlap between adjacent (parallel or anti‐parallel) MTs where it is thought to mediate cross‐linking of bundled MTs, thereby promoting the assembly and stability of linear MT arrays both in vitro and in vivo (Thadani et al., 2009). A role in cross‐linking kinetochore MTs is also observed for TACC3 in HEK293 cells (Booth, Hood, Prior, & Royle, 2011). Given that neuronal MTs are organized into polarized bundles that resemble the MT arrays found in *S. pombe* (Baas, Rao, Matamoros, & Leo, 2016; Bartolini & Gundersen, 2006; Hoogenraad & Bradke, 2009), it seemes possible that dTACC serves a similar function in regulating synaptic MT organization. Interestingly, this possibility would be consistent with findings that Pavarotti, a kinesin that cross‐links MTs and stabilizes the mitotic spindle, is also a negative regulator of NMJ size (McLaughlin, Nechipurenko, Liu, & Broihier, 2016).

### 2.4 TACC is required for normal levels and organization of synaptic MTs

The MT‐lattice localization of dTACC at the NMJ is consistent with previous studies of TACC function in MT organization and stability. In *Drosophila* embryos, loss of dTACC results in short astral and spindle MTs (Gergely, Kidd, et al., 2000), and similar roles in regulating both mitotic and interphase MTs have been observed for TACC proteins in a variety of systems (Ding et al., 2017; Hood and Royle, 2011; Peset and Vernos, 2008; Thakur et al., 2013). Thus, the close association of dTACC with the MT lattice suggested a specific role in regulating MTs within motor axon terminals.

**Figure 4:**
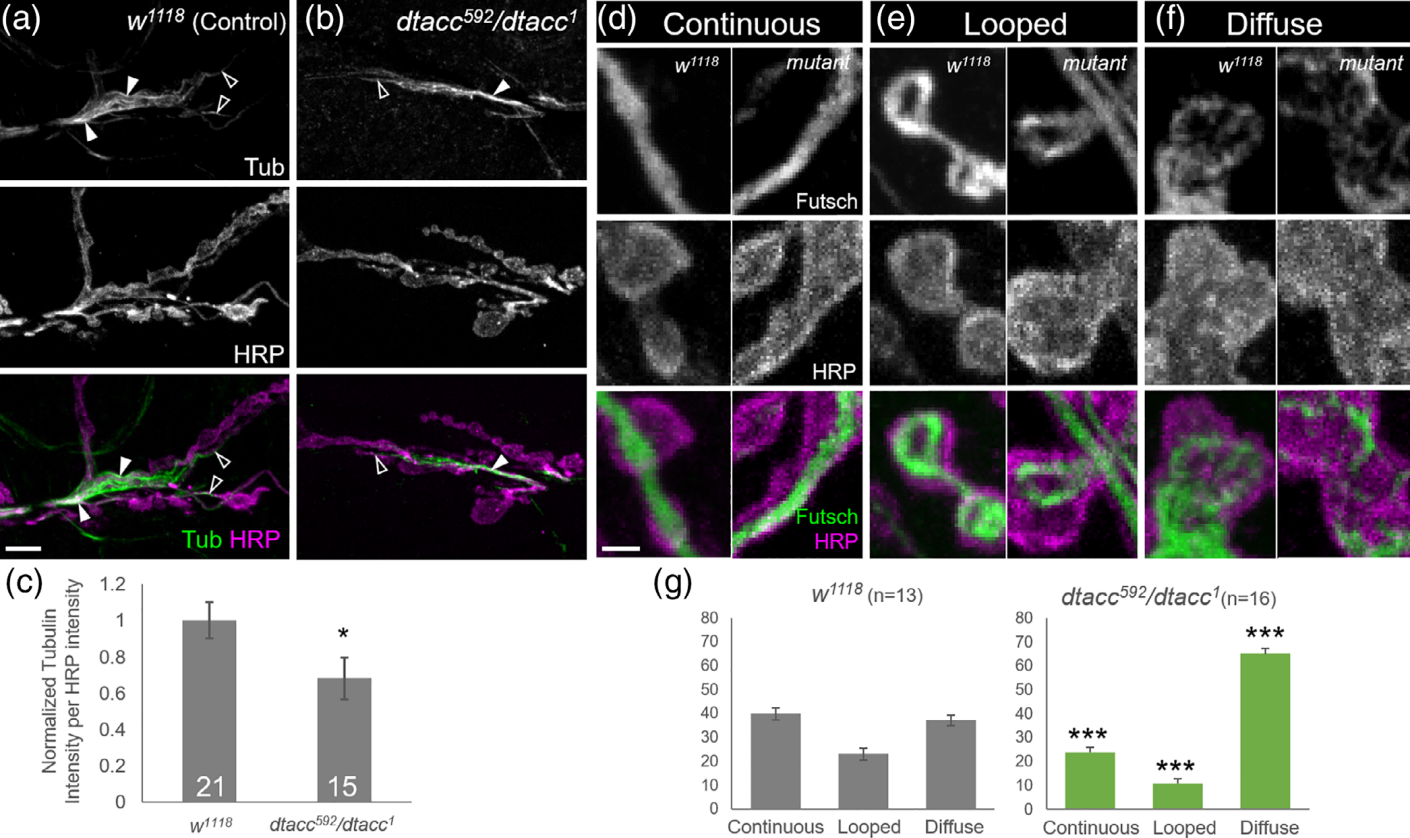
TACC is regulates the architecture and higher‐order organization of synaptic MTs. (a–c) Comparison of tubulin intensity in control and *dtacc* ^*592*^ */dtacc* ^*1*^ NMJs stained with α‐tubulin (Ab15246) and α‐HRP. Control animals (a) showed robust tubulin staining with clear filamentous structures (triangles). Tubulin staining was most concentrated in the main axonal shaft (solid triangles), while thinner tubulin filaments were observed in terminal branches (hollow triangles). In *dtacc* ^*592*^ */dtacc* ^*1*^ flies (b), tubulin staining appeared weaker and was often undetected in branches. Quantification (c) indicates that detectable tubulin intensity was significantly reduced *dtacc* ^*592*^ */dtacc* ^*1*^ flies. Scale bar, 10 μm. (d–g) Analysis of Futsch‐labeled MT arrangements in control and *dtacc* ^*592*^ */dtacc* ^*1*^ NMJs labeled with α‐Futsch (22C10) and α‐HRP. Futsch‐decorated MTs in the three terminal boutons of each branch were categorized into continuous, looped, and diffuse patterns (d–f). Side‐by‐side comparison of boutons showed that Futsch‐MT structures are clearly reproducible between controls and *dtacc* ^*592*^ */dtacc* ^*1*^ mutants. Quantification (g) shows that in *dtacc* ^*592*^ */dtacc* ^*1*^, the distribution of different MT structures is altered, with the frequency of continuous and looped structures very significantly decreased and diffuse staining very significantly increased. *p < .05, ***p < .001, *t*‐test; error bars indicate ± *SEM*; number of NMJs quantified indicated on graph; scale bar, 1 μm

To investigate if dTACC regulates synaptic MTs, we compared the staining intensity and distribution of tubulin in *dtacc* ^*592*^ */dtacc* ^*1*^ animals to *w* ^*1118*^ controls (Figure 4a,b). Due to the fragility of MTs, we used a specifically optimized fixation protocol (see Section 3). Control tubulin staining was clear and robust, with distinct filamentous structures (Figure 4a). There was a clear concentration of tubulin in the main axon shaft, with thinner filaments leading out into the branches of the synaptic terminal where bouton addition is more frequent. In contrast to previous demonstrations (Jin et al., 2009; Mao et al., 2014; Sherwood et al., 2004; Trotta, Orso, Rossetto, Daga, & Broadie, 2004) of robust muscle MT staining, we observed comparatively weaker postsynaptic MT signal. This likely reflects differences in our protocol, which was specifically optimized to target the labile, unstable presynaptic MT population.

Compared to controls, *dtacc* animals displayed diminished or undetectable tubulin staining in terminal branches, where the majority of bouton addition events occur. While tubulin was still detected in the main axon shaft, this population was also visibly reduced (Figure 4b). Consistently, quantification indicated a ~32% overall decrease in tubulin intensity in *dtacc* animals (Figure 4c). This reduction in staining could reflect a decrease in total tubulin mass, however, no obvious decrease in alpha‐tubulin (Ab7291) was detected on our Western blots of *dtacc* mutant larvae (Figure S3A). This suggested that total MT polymer must be reduced in *dtacc* mutants. Alternatively, since single MTs may be difficult to resolve with in vivo light microscopy compared to bundled MT arrays, a change in the spatial organization of MTs could also reduce detectable tubulin staining. This encouraged us to seek an additional histological probe for MTs and their in situ organization.

To further understand correlation between the NMJ size and MT phenotypes, we next tested the effect of dTACC on the formation of higher‐order MT structures that have been associated with different states of bouton growth and division (Conde & Cáceres, 2009; Roos et al., 2000; Ruiz‐Cañada & Budnik, 2006). Most nondividing, *en passant* boutons are traversed by a continuous Futsch‐decorated MT bundle, while nondividing boutons on the ends of branches (i.e., terminal boutons) often display Futsch‐labeled MT loops. Actively growing or dividing terminal boutons display reorganization of loops into dispersed structures, which can appear as diffuse or punctate staining (Conde & Cáceres, 2009; Roos et al., 2000; Ruiz‐Cañada & Budnik, 2006). Given that Futsch binds a subpopulation of stabilized MTs (Roos et al., 2000) and is not known to bind individual tubulin dimers, the diffuse appearance of dispersed/splayed MTs likely reflects structures such as short MT fragments, or longer individual MTs that are splayed from the main bundle, as opposed to free tubulin.

We investigated the effects of *dtacc* loss on Futsch‐labeled MTs structures previously categorized as “continuous,” “looped,” or “diffuse” (Jin et al., 2009; Sherwood et al., 2004). These structures are clearly and reproducibly distinguished in both *w* ^*1118*^ controls and in *dtacc* ^*592*^ */dtacc* ^*1*^ animals (Figure 4d–f). We focused on the three most terminal boutons of each branch, as branch ends are the sites of most active growth (Zito et al., 1999). Loss of *dtacc* produced consistent, measurable changes to Futsch‐MT structures: compared to controls, mutants showed very significantly decreased frequency of both continuous (40% vs. 24%) and loop structures (23% vs. 11%; Figure 4g). In contrast, the majority of *dtacc* boutons (65%) showed diffuse staining, in contrast to controls (37%; Figure 4g). Our findings thus suggest that dTACC promotes the organization of MTs into stable continuous and looped structures in wild‐type, whereas *dtacc* loss nearly doubles the number of boutons containing dispersed/splayed MT structures.

Collectively, our findings suggest that dTACC serves to restrict NMJ overgrowth and regulate MT organization and/or assembly. These studies support the notion that MT regulator is vital to controlling synapse expansion, as implied by studies of several other MT‐associated factors. Interestingly, the frequency of diffuse Futsch staining is increased by 1.8‐fold in *dtacc* mutants (Figure 4g). This is reminiscent of the twofold increase in the number of both mature (Figure 1) and nascent boutons (Figure 2) in *dtacc* animals, and is thus consistent with the possibility that bouton addition and MT organization are linked. A parallel correlation between MT reorganization and membrane growth has been well‐established in the axonal growth cone: MTs are splayed/dispersed in migrating growth cones, and shift to bundled and looped distributions in paused growth cones (Dent, Callaway, Szebenyi, Baas, & Kalil, 1999; Kalil, Szebenyi, & Dent, 2000; Tanaka, Ho, & Kirschner, 1995; Tanaka & Kirschner, 1991). Given the resemblance of the MT organizations we observe at the NMJ (Figure 4d–f) to the distributions of MTs at the growth cone, and the correlation of different MT structures to different growth states in both systems, it seems plausible that the growth cone and synapse share common mechanisms of coupling membrane growth and MTs reorganization despite differences in structure and dynamics.

Our result that the *dtacc* overgrowth phenotype (Figure 1) correlates with a reduction in detectable tubulin (Figure 4a–c), while surprising, is consistent with the loss‐of‐function phenotypes of other MT regulators. For instance, loss of tubulin‐specific chaperone E (*tbce*), which facilitates the folding of α‐tubulin, results in NMJ overgrowth, along with a decrease in both presynaptic Futsch staining and the postsynaptic MT network (Jin et al., 2009). Intriguingly, the overgrowth phenotype of *spastin* mutants is also accompanied by a reduction in both tubulin and Futsch staining, despite the function of Spastin as a MT‐destabilizer (Sherwood et al., 2004). It has been proposed that the MT severing activity of Spastin generates seeds that nucleate the growth of new MTs (Roll‐Mecak & Vale, 2006), thus explaining the attenuated MT network of *spastin* mutants (Sherwood et al., 2004). Collectively, our findings in *dtacc* mutants, as well as the previous studies of *tbce* and *spastin*, suggest that NMJ growth may not be correlated solely with MT stabilization and levels, but may also be related to the organization of MTs. This possibility is consistent with prior studies that have shown a correlation of displayed/splayed MT structures with actively growing or dividing boutons (Conde & Cáceres, 2009; Roos et al., 2000; Ruiz‐Cañada & Budnik, 2006). Indeed, both *tbce* and *spastin* mutants show increases in diffuse Futsch‐MT staining concurrent with NMJ overgrowth (Jin et al., 2009; Sherwood et al., 2004).

In conclusion, we demonstrate that dTACC is a negative regulator of bouton addition during the development of the NMJ and that dTACC associates with and regulates the stability and organization of synaptic MTs. We provide evidence that dTACC promotes MT structures associated with paused bouton growth and division. Further studies may investigate the functional partners of dTACC at the NMJ, and how the roles of dTACC may relate to the roles of factors such as TBCE and Spastin, which show similar overgrowth and MT organization phenotypes.

## 3 MATERIALS AND METHODS

### 3.1 Drosophila genetics

Stocks were raised at 25°C according to standard procedures. The *w* ^*1118*^, *elaV* ^*C155*^ *‐*GAL4, *UAS‐Dcr2*, *OK6*‐GAL4, and *Df(3R)110* stocks were obtained from the Bloomington Stock Center (Bloomington, IN). The *UAS‐dtacc‐RNAi* stock was obtained from the Vienna *Drosophila* Resource Center (Vienna, Austria). To enhance *dtacc‐RNAi* expression, *elaV* ^*C155*^ *‐*GAL4 was also used to express *UAS‐Dcr2*, an endonuclease that promotes processing of long dsRNAs to siRNAs. The previously described *msps* ^*P*^ (Cullen, Deák, Glover, & Ohkura, 1999), *dtacc* ^*1*^ (Gergely, Kidd, et al., 2000), and *dtacc* ^*592*^ (Lee et al., 2001) stocks were provided by Jordan Raff.

### 3.2 Antibody production and purification

dTACC sequence containing amino acids 146–327 was His‐tagged, bacterially expressed, and purified. dTACC antibody was raised in mice against and purified by PrimmBiotech, Inc. (Cambridge, MA).

### 3.3 Immunohistochemistry

First instars and wandering third instars were dissected in Ca^2+^‐free saline and fixed in 4% paraformaldehyde in PBS for 10 min, except for tubulin immunostaining, where larvae were dissected in Brinkley Buffer 1980 (80 mM PIPES, 1 mM MgCl2, 1 mM EGTA, pH 6.8) and fixed in 4% paraformaldehyde in PBS with 5 mM EGTA. Primary antibodies obtained from the Developmental Studies Hybridoma Bank (Iowa City, IA) include: mouse anti‐Brp NC82 (1:50), mouse anti‐Dlg 4F3 (1:50), and mouse anti‐Futsch (1:50). The following primary antibodies were also used for immunohistochemistry: mouse anti‐dTACC (1:50) and rabbit anti‐alpha‐tubulin (1:200; Ab15246; Abcam, Cambridge, UK). Secondary antibodies conjugated to AlexaFluor 488 and 594 were used (1:200; Invitrogen, Waltham, MA. USA). Anti‐HRP antibodies conjugated to AlexaFluor 594 and 647 were used (1:200; Jackson Immunoresearch, West Grove, PA, USA).

### 3.4 Activity assay

The spaced‐stimulation paradigm was adapted from published protocols (Ataman et al., 2008; Nesler et al., 2013; Piccioli & Littleton, 2014). Larvae were semidissected in HL3 (in mM): 70 NaCl, 5 KCl, 0.2 CaCl_2_, 20 MgCl_2_, 10 NaHCO_3_, 5 trehalose, 115 sucrose, 5 HEPES‐NaOH, pH 7.2. Relaxed filets were pulsed with four 5 min 25°C incubations with high K^+^ solution (in mM): 40 NaCl, 90 KCl, 1.5 CaCl_2_, 20 MgCl_2_, 10NaHCO_3_, 5 trehalose, 5 sucrose, and 5 HEPES‐NaOH, pH 7.2, spaced by 15 min in 25°C HL3. After the fourth high K^+^ pulse, larvae were allowed to recover in HL3 solution for 15 min, stretched, and then fixed. Nascent boutons were identified by lack of Brp or Dlg staining.

### 3.5 Western blotting

See Supporting Information.

### 3.6 Image acquisition and analysis

Synaptic arbors of muscle 6/7 in the abdominal segment A2 were used for all analyses. Imaging was performed on a Nikon A1R point scanning confocal and a Nikon Yokogawa spinning disc confocal with a Hamamatsu ORCA‐R2 cooled CCD camera. 3D‐SIM was performed on a DeltaVision OMX Blaze microscope (GE Healthcare Life Sciences, Marlborough, MA, USA) with a PCO sCMOS camera. Lasers were adjusted to prevent oversaturation. Images were processed and analyzed with ImageJ and/or MATLAB. Bouton number and size were counted and traced by hand. An HRP mask was used to restrict analysis to neuronal signal for intensity analysis, and MATLAB scripts were used to quantify dTACC and tubulin signals relative to HRP. Line scans were used to create intensity profiles to distinguish different Futsch structures.

### 3.7 Statistics

All comparisons were done using Welch's *t*‐test for unequal variances using Graphpad.

## AUTHOR CONTRIBUTIONS

Conceptualization: V.T.C., S.J., J.L., D.V.V.; Methodology: V.T.C., D.V.V.; Formal analysis: V.T.C., S.J.; Investigation: V.T.C., S.J., J.L., M.V.; Resources: V.T.C., S.J., J.L., D.V.V.; Data curation: V.T.C.; Writing—Original draft: V.T.C., D.V.V.; Writing—Review and editing: V.T.C., S.J., D.V.V.; Visualization: V.T.C., D.V.V.; Funding acquisition: V.T.C., D.V.V.; Supervision: D.V.V.

## Supporting information


**Figure S1** Confirmation of TACC null alleles and first‐instar phenotype. (A) *dtacc* ^*592*^ */Df(3R)110* and *dtacc* ^*1*^ */Df(3R)110* flies were generated. Both individual TACC alleles produced overgrowth compared to *w* ^*1118*^ controls. (B–D) *dtacc* phenotype in first‐instar animals. Compared to controls (B), *dtacc* ^*1*^ animals (C) showed significant overgrowth, as confirmed by quantification (D). ***p < .001, determined by Student's *t*‐test; error bars indicate ± *SEM*; number of NMJs quantified indicated on graph; scale bar, 5 μm.
**Figure S2**. *dtacc* animals show normal accumulation of pre‐ and postsynaptic markers. *w* ^*1118*^ and *dtacc* ^*592*^ */dtacc* ^*1*^ animals were costained with the neuronal membrane marker α‐HRP and the presynaptic active zone marker α‐Brp (A and B) or the postsynaptic marker α‐Dlg (C and D). Compared to *w* ^*1118*^ (A and C) animals, the distribution of markers in *dtacc* ^*592*^ */dtacc* ^*1*^ (B and D) animals was grossly normal. Scale bar, 5 μm.
**Figure S3**. Validation of the TACC antibody. (A) Western blotting showed complete reduction of dTACC antibody signal in *dtacc* ^*592*^ */dtacc* ^*1*^ null animals. α‐alpha‐tubulin (Ab7291) was used as a loading control. (B) dTACC staining intensity was significantly reduced in *dtacc* null flies by ~68%. ***p < .001, determined by Student's *t*‐test; error bars indicate ± *SEM*; number of NMJs quantified indicated on graph. (C–E) First‐instar *w* ^*1118*^ animals were costained with α‐dTACC and α‐HRP. (C) Schematic showing dissection technique of first instars, which removes the brain lobes but leaves the ventral nerve cord (VNC) intact. Strong dTACC staining was observed in the VNC (D) and throughout the motor and sensory axon tracts (E; triangles), along with some muscle staining. Scale bars, 50 μm.

## Data Availability

The data that support the findings of this study are available from the corresponding author upon reasonable request.
