# Supplementary material for: dTACC restricts bouton addition and regulates microtubule organization at the Drosophila neuromuscular junction
Source: Cytoskeleton (Hoboken). 2019 Nov 21;77(1-2):4–15. doi: 10.1002/cm.21578 (PMC7027520; doi:10.1002/cm.21578)

**SUPPLEMENTAL INFORMATION**

**SUPPLEMENTAL MATERIALS AND METHODS**

**Western blotting**

30 wandering third-instar larvae of each genotype were dissected in ice-cold dissection buffer (PBS, 1 mM EGTA, 1× cOmplete, Mini Protease Inhibitor Cocktail; Roche), leaving body wall musculature. Dissected pelts were homogenized in lysis buffer (dissection buffer, 0.5% Tween-20). Homogenates were loaded onto 4-15% SDS-PAGE gels (BioRad). Protein was transferred to PVDF membrane (BioRad) and immunoblotted using standard protocols and exposed using chemiluminescence reagents (Thermo Scientific). The following antibodies were used for blotting: mouse anti-TACC (1:50), mouse anti-alpha tubulin (1:5000; Ab7291; Abcam) goat anti-mouse HRP (1:1000; Cell Signaling Technology).

**SUPPLEMENTAL FIGURE LEGENDS**

**Figure S1. Confirmation of TACC null alleles and first instar phenotype. *A*,** *dtacc^592^/Df(3R)110* and *dtacc^1^/ Df(3R)110* flies were generated. Both individual TACC alleles produced overgrowth compared to *w^1118^* controls. ***B-D***, *dtacc* phenotype in first-instar animals. Compared to controls (**B**), *dtacc^1^* animals (**C**) showed significant overgrowth, as confirmed by quantification (**D**). *** P < 0.001, determined by Student’s t-test; error bars indicate ± s.e.m; number of NMJs quantified indicated on graph; scale bar, 5 µm.

**Figure S2. *dtacc* animals show normal accumulation of pre- and postsynaptic markers.** *w^1118^* and *dtacc^592^/dtacc^1^* animals were co-stained with the neuronal membrane marker α-HRP and the presynaptic active zone marker α-Brp (**A,B**) or the postsynaptic marker α-Dlg (**C,D**). Compared to *w^1118^* (**A,C**) animals, the distribution of markers in *dtacc^592^/dtacc^1^* (**B,D**) animals was grossly normal. Scale bar, 5 µm.

**Figure S3. Validation of the TACC antibody. *A*,** Western blotting showed complete reduction of dTACC antibody signal in *dtacc^592^/dtacc^1^* null animals. α-alpha-tubulin (Ab7291) was used as a loading control. ***B***, dTACC staining intensity was significantly reduced in *dtacc* null flies by ~68%. *** P < 0.001, determined by Student’s t-test; error bars indicate ± s.e.m; number of NMJs quantified indicated on graph. ***C-E***, First instar *w^1118^* animals were co-stained with α-dTACC and α-HRP. ***C***, Schematic showing dissection technique of first instars, which removes the brain lobes but leaves the ventral nerve cord (VNC) intact. Strong dTACC staining was observed in the VNC (**D**) and throughout the motor and sensory axon tracts (**E**; triangles), along with some muscle staining. Scale bars, 50 µm.

**Figure S1**


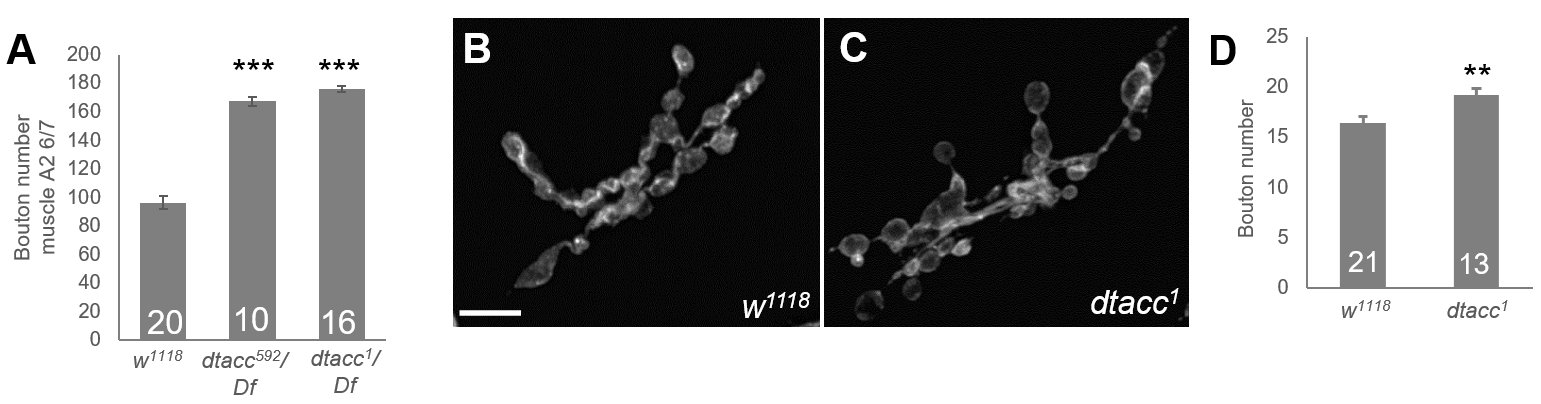


**Figure S2**


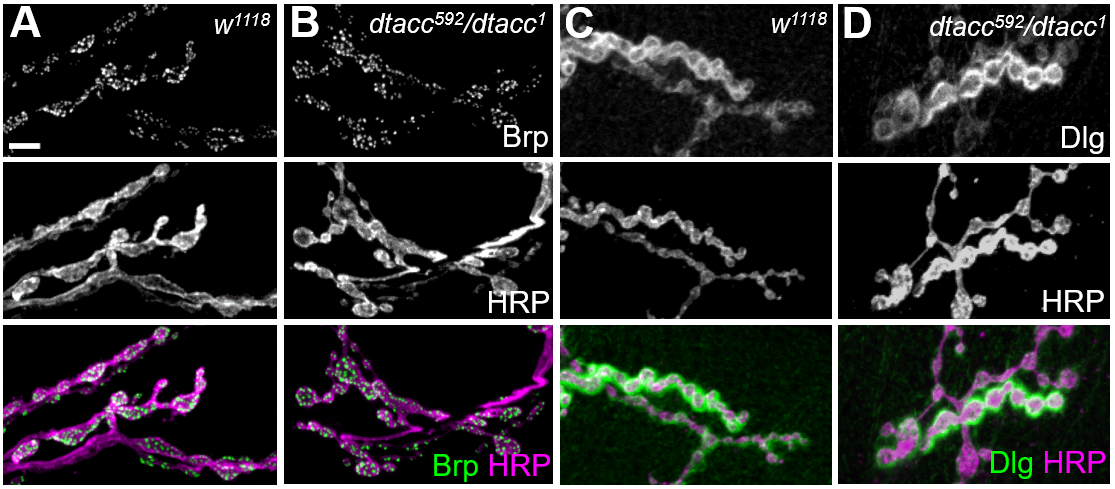


**Figure S3**


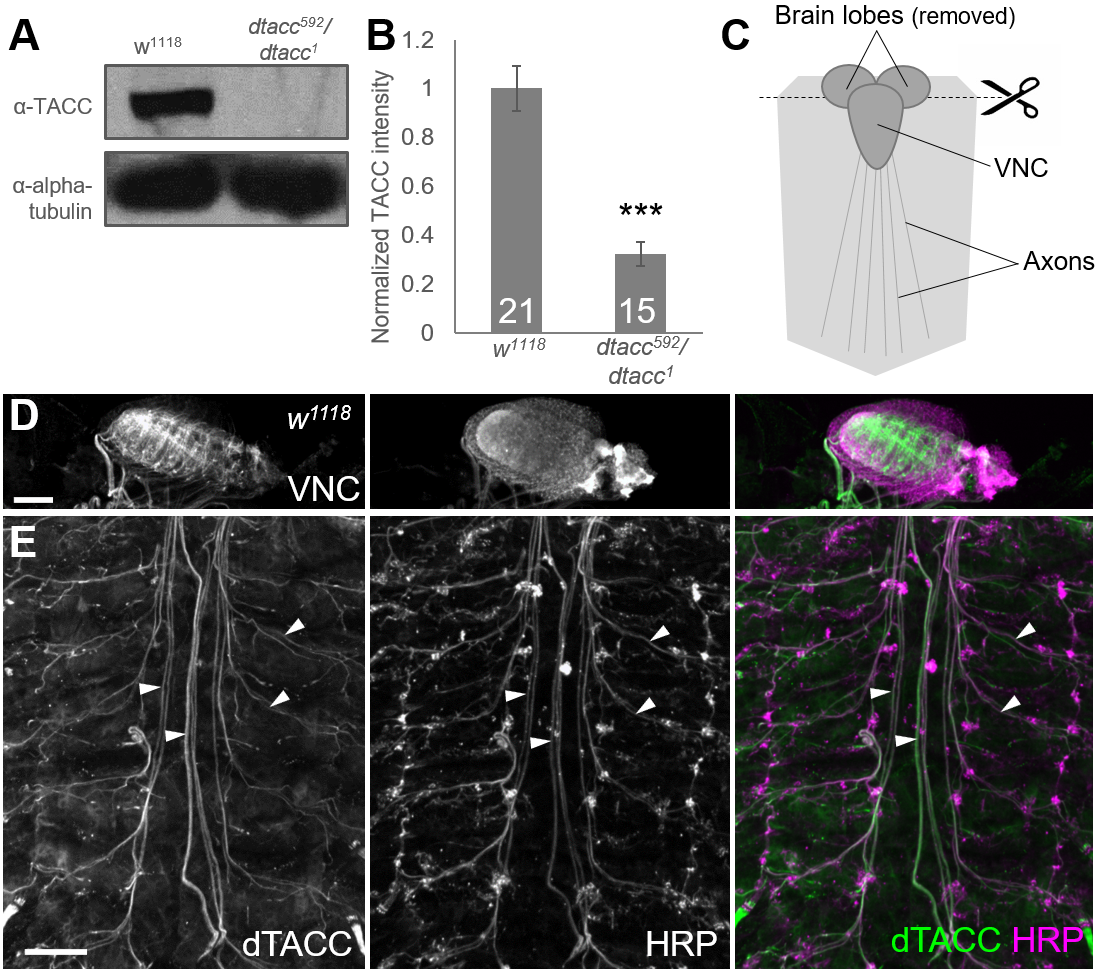

Supplement: Supplementary file 1 — Figure S1 Confirmation of TACC null alleles and first‐instar phenotype. (A) dtacc 592 /Df(3R)110 and dtacc 1 /Df(3R)110 flies were generated. Both individual TACC alleles produced overgrowth compared to w 1118 controls. (B–D) dtacc phenotype in first‐instar animals. Compared to controls (B), dtacc 1 animals (C) showed significant overgrowth, as confirmed by quantification (D). ***p < .001, determined by Student's t‐test; error bars indicate ± SEM; number of NMJs quantified indicated on graph; scale bar, 5 μm. Figure S2. dtacc animals show normal accumulation of pre‐ and postsynaptic markers. w 1118 and dtacc 592 /dtacc 1 animals were costained with the neuronal membrane marker α‐HRP and the presynaptic active zone marker α‐Brp (A and B) or the postsynaptic marker α‐Dlg (C and D). Compared to w 1118 (A and C) animals, the distribution of markers in dtacc 592 /dtacc 1 (B and D) animals was grossly normal. Scale bar, 5 μm. Figure S3. Validation of the TACC antibody. (A) Western blotting showed complete reduction of dTACC antibody signal in dtacc 592 /dtacc 1 null animals. α‐alpha‐tubulin (Ab7291) was used as a loading control. (B) dTACC staining intensity was significantly reduced in dtacc null flies by ~68%. ***p < .001, determined by Student's t‐test; error bars indicate ± SEM; number of NMJs quantified indicated on graph. (C–E) First‐instar w 1118 animals were costained with α‐dTACC and α‐HRP. (C) Schematic showing dissection technique of first instars, which removes the brain lobes but leaves the ventral nerve cord (VNC) intact. Strong dTACC staining was observed in the VNC (D) and throughout the motor and sensory axon tracts (E; triangles), along with some muscle staining. Scale bars, 50 μm. [file CM-77-4-s001.docx]
